# Exosomes as Disease-Informed Nanoplatforms for Pulmonary Fibrosis: From Pathogenic Signaling to Precision Diagnosis and Therapy

**DOI:** 10.3390/pharmaceutics18060668

**Published:** 2026-05-28

**Authors:** Jeong Min Lee, Kyung Tae Kim, Chung-Sung Lee, Hee Sook Hwang

**Affiliations:** 1 Department of Pharmaceutical Engineering, Dankook University, Cheonan 31116, Republic of Korea; 2 Department of Pharmaceutical Engineering, Soonchunhyang University, Asan 31538, Republic of Korea; 3 Institute for Molecular Metabolism Innovation, Soonchunhyang University, Asan 31538, Republic of Korea

**Keywords:** pulmonary fibrosis, exosomes, nanotherapeutics, drug delivery, precision medicine

## Abstract

Pulmonary fibrosis (PF) is a progressive and often fatal interstitial lung disease for which the currently available pharmacological therapies remain largely limited to slowing disease progression rather than reversing established fibrosis. This limitation has stimulated increasing interest in innovative therapeutic platforms capable of modulating complex fibrotic pathways. In this context, exosomes—nanoscale extracellular vesicles—have emerged as promising cell-free nanocarriers due to their intrinsic biocompatibility, low immunogenicity, and ability to be engineered for targeted drug delivery. In this review, we provide a comprehensive overview of both natural and engineered exosome-based strategies for the diagnosis and treatment of pulmonary fibrosis. We summarize recent advances in exosome engineering, including ligand functionalization, glycoengineering, and therapeutic cargo loading, highlighting how these approaches may support the development of more targeted and potentially personalized nanotherapeutic strategies. We further discuss emerging hybrid delivery platforms, such as exosome–liposome chimeras and hydrogel-based depots, which may enhance pulmonary retention, improve therapeutic durability, and enable controlled drug release. Finally, we outline key challenges and opportunities for clinical translation, including large-scale manufacturing, regulatory considerations, and clinically relevant delivery routes such as inhalation-based administration. Collectively, this review provides a translational perspective on engineered exosomes as emerging nanotherapeutic platforms for pulmonary fibrosis.

## 1. Introduction

Pulmonary fibrosis (PF) encompasses a heterogeneous group of interstitial lung disorders characterized by excessive extracellular matrix (ECM) deposition, fibroblast proliferation, and irreversible distortion of alveolar architecture [1,2]. These include idiopathic pulmonary fibrosis (IPF), connective tissue disease-associated PF, occupational or environmental fibrotic lung disease, and post-injury fibrotic disorders. IPF, the most prevalent subtype, carries a median survival of only 3–5 years after diagnosis, underscoring the urgent need for more effective therapeutic strategies [3,4,5]. Patients with IPF frequently present with multiple comorbidities, including pulmonary hypertension, gastroesophageal reflux disease, obesity, emphysema, cardiovascular disease, and obstructive sleep apnea, all of which further complicate disease management and worsen clinical outcomes [6]. Although most patients experience a gradual decline in lung function, approximately 10–15% undergo rapid disease acceleration within months, often culminating in acute respiratory failure [7]. Accurate estimates of IPF incidence and prevalence remain challenging due to diagnostic complexity, frequent misclassification, and inconsistencies in clinical coding practices [8]. Together, IPF and its associated comorbidities substantially impair health-related quality of life and impose a considerable socioeconomic burden [9].

Current clinical diagnosis of PF relies on multidisciplinary evaluation integrating high-resolution computed tomography (HRCT), pulmonary function testing, clinical history, and in selected cases, histopathological assessment [10]. Despite advances in disease management, currently approved anti-fibrotic agents—pirfenidone and nintedanib—provide only modest clinical benefit and do not reverse established fibrosis [11,12]. Both agents are approved by the European Medicines Agency and the U.S. Food and Drug Administration and are recommended in international treatment guidelines [13,14]. In Phase 3 clinical trials, including ASCEND (NCT01366209) and CAPACITY (NCT00287729, NCT00287716), pirfenidone reduced the risk of disease progression or death compared with the placebo, while nintedanib demonstrated similar efficacy in the INPULSIS trials (NCT01335464, NCT01335477). Nevertheless, neither therapy halts disease progression in most patients, and treatment is frequently limited by gastrointestinal and hepatic adverse effects, poor tolerability, and high cost, leading to dose reduction or discontinuation in real-world settings [15,16,17]. As a result, current pharmacological approaches primarily delay functional decline rather than fundamentally altering the disease course.

Importantly, the limited efficacy of existing therapies reflects not only pharmacological constraints but also a fundamental mismatch between the multifactorial pathobiology of PF and single-target therapeutic paradigms. Fibrotic progression arises from the dynamic interplay of epithelial injury, immune dysregulation, fibroblast activation, and aberrant wound-healing responses, rendering isolated pathway inhibition insufficient for durable disease control [18,19]. This complexity has contributed to repeated translational failures, in which interventions showing promise in preclinical models fail to achieve meaningful clinical benefit. Furthermore, the lack of reliable biomarkers for early diagnosis, disease stratification, and treatment monitoring continues to hinder timely intervention and the development of personalized therapeutic strategies [20,21,22].

Collectively, these limitations have driven growing interest in alternative therapeutic modalities capable of addressing multiple pathological processes simultaneously. In this context, cell-free nanoscale systems—particularly extracellular vesicles (EVs) and exosomes (EXOs)—have emerged as attractive candidates, offering regenerative, immunomodulatory, and anti-fibrotic potential while avoiding many of the safety concerns associated with whole-cell transplantation [23,24,25].

EXOs are nanoscale extracellular vesicles (30–150 nm) originating from the endosomal compartment of most eukaryotic cells and serve as key mediators of intercellular communication under both physiological and pathological conditions [26,27,28]. Due to their endogenous origin, EXOs exhibit high biocompatibility, low intrinsic toxicity, and prolonged stability in circulation, partly through their ability to evade immune surveillance. These properties distinguish EXOs from many synthetic nanocarriers, whose clinical translation is often limited by rapid clearance, immunogenicity, or off-target toxicity [29,30,31].

Functionally, EXOs transport a diverse repertoire of bioactive cargos—including proteins, lipids, messenger RNAs, and non-coding RNAs such as microRNAs and long non-coding RNAs—that reflect the physiological state and identity of their parent cells [32,33,34]. Upon uptake by recipient cells, EXOs can reprogram cellular behavior by modulating gene expression, signaling pathways, and metabolic states, thereby contributing to tissue homeostasis or disease progression in a context-dependent manner [35,36,37]. These intrinsic signaling capabilities have positioned EXOs as promising therapeutic agents in regenerative medicine and immunomodulation, particularly in diseases characterized by chronic inflammation, fibrosis, and impaired tissue repair [38,39,40].

Compared with cell-based therapies, EXO-based strategies offer key safety and translational advantages, including reduced risk of immune rejection, uncontrolled engraftment, and tumorigenicity, while retaining beneficial paracrine signaling functions [41,42,43]. Their nanoscale size and amenability to bioengineering further support their utility as therapeutic and diagnostic platforms [44,45]. These features have stimulated growing interest in EXO-based strategies for PF, where preclinical studies suggest potential anti-fibrotic, immunomodulatory, and regenerative effects [46].

Importantly, however, not all EXOs are inherently therapeutic. Their biological effects are highly context-dependent and governed by factors such as cellular origin, disease stage, microenvironmental cues, and cargo composition. Without rigorous mechanistic insight and rational design, exosome-based interventions may inadvertently propagate pathological signaling or reproduce translational challenges previously encountered in nanomedicine. Accordingly, the successful clinical development of EXO-based therapeutics will require a shift from empirical application toward mechanism-driven engineering and precise functional characterization. In addition, therapeutic EXOs may also present potential safety concerns, including unintended activation of off-target signaling pathways, immune recognition following repeated administration, or variability arising from donor cell source and cargo heterogeneity. These considerations highlight the importance of rigorous safety evaluation, standardized characterization, and careful engineering design for clinical translation.

The rationale for applying EXO-based strategies to PF lies in their ability to modulate multiple interconnected pathological processes, including inflammation, fibroblast activation, and aberrant tissue remodeling [47,48,49,50,51]. In addition to their intrinsic biological activity, advances in exosome engineering have expanded their potential as targeted therapeutic delivery platforms.

These mechanistic advantages are supported by growing preclinical evidence demonstrating that EXOs derived from MSCs or macrophages can attenuate fibrotic pathology in experimental PF models, including reduced collagen deposition, partial restoration of alveolar architecture, and the suppression of pro-inflammatory cytokines such as TNF-α and IL-6 [52]. Beyond their intrinsic biological activity, EXOs also offer favorable delivery characteristics, including high biocompatibility, circulatory stability, and compatibility with both systemic and local administration routes [53,54]. Furthermore, advances in exosome engineering—including surface functionalization, therapeutic cargo loading, and hybridization with synthetic nanocarriers—have further expanded their potential as targeted anti-fibrotic delivery platforms (Table 1) [55,56,57].

Despite rapid advances in the field, however, EXO-based research in PF remains conceptually fragmented. Diagnostic applications, therapeutic studies, and engineering strategies are often pursued in parallel but discussed in isolation, frequently without sufficient integration of disease biology, biomarker discovery, and translational constraints. As a result, promising experimental findings do not always converge toward clinically actionable strategies.

**Figure 1:**
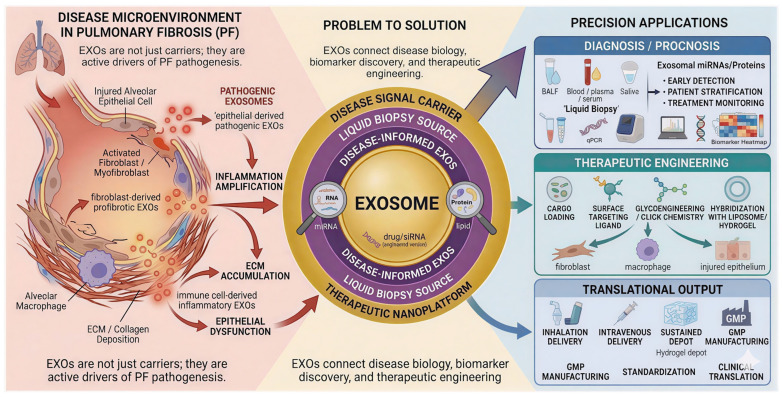
Exosomes as disease-informed nanoplatforms in pulmonary fibrosis. In pulmonary fibrosis (PF), exosomes (EXOs) released from injured epithelial cells, activated fibroblasts, and immune cells propagate profibrotic signaling, amplify inflammation, and promote extracellular matrix deposition within the fibrotic lung microenvironment. At the same time, EXOs isolated from biofluids such as bronchoalveolar lavage fluid, blood, and saliva serve as accessible liquid-biopsy platforms carrying disease-relevant molecular signatures, including microRNAs, proteins, and other regulatory cargos. Through rational engineering strategies, such as cargo loading, surface functionalization, glycoengineering, and hybridization with synthetic carriers or biomaterials, EXOs can be repurposed into targeted anti-fibrotic nanotherapeutics. Collectively, EXOs bridge PF pathogenesis, biomarker discovery, and targeted therapeutic development, highlighting their potential as integrated platforms for diagnosis, treatment, and clinical translation.

In this review, we seek to address this gap by proposing a unified framework that conceptualizes EXOs not merely as therapeutic carriers, but as disease-informed nanoplatforms that bridge biomarker identification, mechanistic insight, and the development of more targeted therapeutic strategies (Figure 1). We first summarize the biological characteristics and fibrogenic roles of EXOs in pulmonary disease, followed by an evaluation of their emerging utility as diagnostic and prognostic biomarkers. We then critically examine therapeutic EXOs derived from MSCs and other cellular sources, as well as engineered and hybrid EXO platforms designed to enhance targeting efficiency and anti-fibrotic efficacy. Finally, we discuss key translational considerations—including manufacturing scalability, delivery strategies, and regulatory challenges—to delineate which EXO-based approaches are most likely to achieve clinical relevance and which may require fundamental rethinking to overcome current limitations.

## 2. EXOs in PF: Biology and Pathogenic Role

### 2.1. Biogenesis, Composition, and Cargo Sorting of EXOs

**Figure 2:**
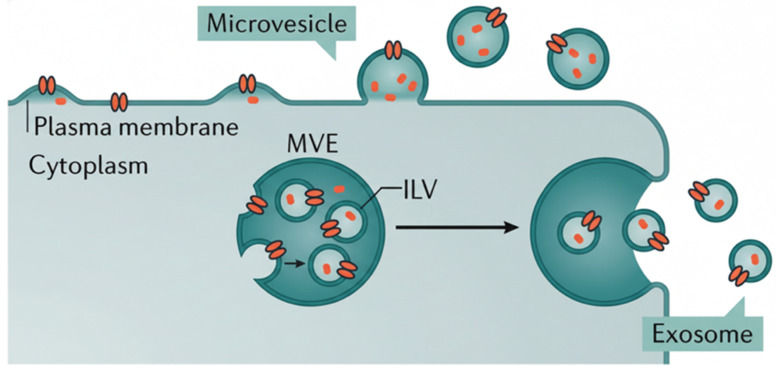
Biogenesis pathways of exosomes (EXOs). Microvesicles are generated by direct outward budding and fission of the plasma membrane, whereas EXOs originate from the endosomal pathway through the formation of intraluminal vesicles (ILVs) within multivesicular endosomes (MVEs), followed by release into the extracellular space upon the fusion of MVEs with the plasma membrane. Adapted from Ref. [64].

EXOs are nanoscale EVs (30–150 nm) generated through the endosomal pathway, and their biogenesis constitutes a tightly regulated process that fundamentally shapes vesicle composition and function [27]. EXO biogenesis proceeds through the endosomal trafficking pathway, in which endocytosed membrane components are processed through early and late endosomal compartments, ultimately leading to the formation of multivesicular bodies (MVBs) [60,61,62]. Within MVBs, membrane invagination gives rise to intraluminal vesicles (ILVs) that selectively incorporate proteins, lipids, and nucleic acids reflective of the cellular state. Upon the fusion of MVBs with the plasma membrane, ILVs are released into the extracellular space as EXOs (Figure 2). This process is governed by both endosomal sorting complexes required for transport (ESCRT)-dependent and ESCRT-independent mechanisms, involving ceramides and tetraspanins (e.g., CD9, CD63, and CD81), while Rab GTPases such as Rab27a/b and Rab11 regulate MVB trafficking and vesicle release [63,64,65,66,67,68,69,70]. Importantly, the molecular decisions made during EXO biogenesis provide a mechanistic basis for the marked heterogeneity observed in EXO cargo and downstream biological effects.

The molecular composition of EXOs is partly conserved yet highly context-dependent. While EXOs characteristically express canonical surface markers such as CD63, CD81, and TSG101, their molecular cargo varies substantially according to the originating cell type and its physiological or pathological state [71,72,73]. EXOs transport a broad spectrum of functional biomolecules including structural proteins (e.g., actin and tubulin), heat shock proteins (e.g., Hsp70 and Hsp90), signaling molecules (e.g., STAT3 and kinases), enzymes, and adhesion-related proteins [74,75]. Notably, their nucleic acid cargo has attracted considerable attention, as EXOs selectively package messenger RNAs (mRNAs), microRNAs (miRNAs), long non-coding RNAs (lncRNAs), and circular RNAs (circRNAs), which can exert profound regulatory effects on gene expression in recipient cells [76,77,78].

The selective sorting of RNA into EXOs is mediated by sequence motifs, RNA-binding proteins such as hnRNPA2B1, and lipid raft-associated mechanisms, enabling targeted and functionally relevant cargo delivery [79,80,81]. By encapsulating and protecting these biomolecules, EXOs enhance their stability and bioavailability, thereby facilitating effective intercellular communication following uptake by recipient cells [82]. Consistent with this role, exosomal miRNAs and lncRNAs have been implicated in the pathogenesis of multiple lung diseases. For example, exosomal *miR-21* derived from human bronchial epithelial cells exposed to cigarette smoke extract promotes fibroblast-to-myofibroblast differentiation in chronic obstructive pulmonary disease [83,84,85], while lncRNAs such as *H19* and *MEG3* have been reported to be upregulated in patients with IPF, highlighting their potential utility as disease-associated biomarkers [86]. In addition, exosomal protein cargos can exert potent biological effects, as demonstrated by vimentin-containing EXOs derived from metastatic lung cancer cells or end-stage lung cancer serum, which induce epithelial–mesenchymal transition in bronchial epithelial cells [87].

In the context of PF, however, EXO cargo has emerged as a critical determinant of pathological intercellular communication. EXOs released by injured alveolar epithelial cells or activated fibroblasts are frequently enriched in profibrotic cargos such as *miR-21*, *miR-199a-5p*, and TGF-β1, which collectively promote myofibroblast differentiation, ECM deposition, and inflammatory activation in neighboring cells. These observations provide a mechanistic basis for how chronic epithelial injury and aberrant repair responses propagate fibrotic signaling beyond the initially damaged regions of the lung.

Taken together, these findings underscore the context-dependent nature of exosome biology in PF, where EXO-mediated signaling may either exacerbate or mitigate fibrotic progression depending on the cellular source and cargo composition. EXOs released from injured epithelial cells, activated fibroblasts, or pro-inflammatory immune cells frequently propagate a fibrotic microenvironment by reinforcing profibrotic signaling loops among epithelial, stromal, and immune compartments [88,89,90]. In contrast, EXOs derived from MSCs, endothelial progenitor cells (EPCs), or immunomodulatory macrophages tend to exert anti-fibrotic effects through the delivery of regulatory miRNAs (e.g., *miR-29*, *miR-let-7d*, and *miR-30*), growth factors such as hepatocyte growth factor and keratinocyte growth factor, and immunomodulatory proteins that collectively suppress fibroblast activation and promote tissue repair [91,92,93,94,95].

This duality highlights a central concept: the biological outcome of EXO-mediated signaling is dictated not by the vesicle itself, but by its cellular origin, cargo composition, and disease context. Accordingly, a detailed understanding of EXO biogenesis, molecular composition, and cargo sorting mechanisms is essential not only for elucidating fibrotic pathogenesis, but also for the rational development of engineered EXOs as therapeutic agents and the identification of vesicle-based biomarkers that reflect disease stage and treatment response [4,61].

### 2.2. EXO-Mediated Regulation of PF

**Figure 3:**
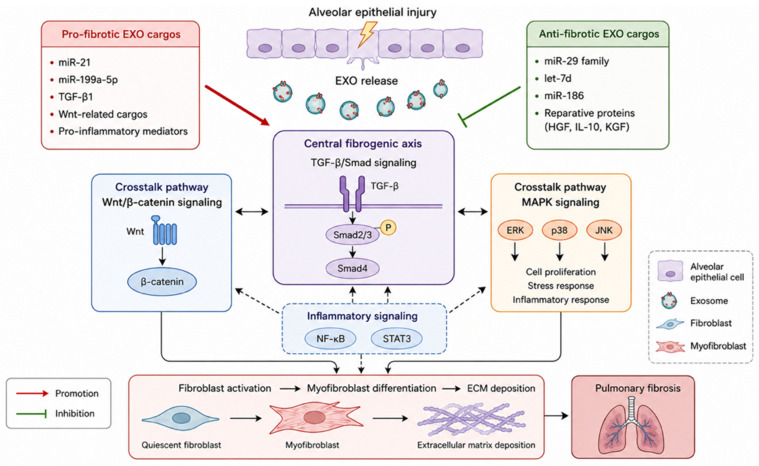
EXO-mediated signaling hierarchy in pulmonary fibrosis. Schematic overview of the major signaling pathways involved in EXO-mediated regulation of pulmonary fibrosis. TGF-β/Smad signaling is presented as the central fibrogenic axis, with reciprocal crosstalk involving the Wnt/β-catenin, MAPK, and inflammatory signaling pathways. The figure further illustrates how distinct pro-fibrotic and anti-fibrotic EXO cargos influence fibroblast activation, myofibroblast differentiation, and extracellular matrix deposition.

The ECM provides essential structural and biochemical cues that regulate tissue homeostasis, but its persistent accumulation is a defining pathological feature of PF [96]. In the fibrotic lung, unresolved epithelial injury and aberrant wound-healing responses lead to sustained myofibroblast activation and excessive ECM deposition, thereby establishing a self-perpetuating fibrotic niche [97]. These processes are governed by interconnected signaling networks, among which transforming growth factor-β (TGF-β)/Smad signaling serves as the canonical central fibrogenic axis, with the Wnt/β-catenin, MAPK, and inflammatory signaling pathways acting as interconnected modulatory networks (Figure 3) [98,99,100,101,102,103].

Increasing evidence indicates that EXOs function as critical intermediaries within these signaling networks by enabling direct intercellular transfer of fibrogenic or anti-fibrotic cues [104,105]. In PF, chronic epithelial damage initiates sustained crosstalk among alveolar epithelial cells, fibroblasts, endothelial cells, and immune cells, a process that is increasingly recognized to be mediated by EXO-based communication [106,107]. Through the delivery of functional RNAs, proteins, and lipids, EXOs modulate recipient cell phenotypes and amplify or restrain fibrogenic signaling cascades within the lung microenvironment [59,108,109].

Pro-fibrotic EXOs are commonly derived from injured epithelial cells, activated fibroblasts, and pro-inflammatory macrophages [24,110]. For example, epithelial cell-derived EXOs released following TGF-β1 stimulation carry *miR-21*, *miR-199a-5p*, and Wnt pathway components that are readily internalized by lung fibroblasts [85]. These cargos activate Smad3-dependent transcription, promote α-smooth muscle actin (α-SMA) expression, and enhance ECM production, including collagen type I and fibronectin [111,112]. In parallel, fibroblast-derived EXOs under fibrotic conditions reinforce autocrine and paracrine activation loops, while EXOs from M1-like macrophages exacerbate fibrosis by transferring inflammatory cytokines and regulatory RNAs that activate NF-κB and STAT3 signaling in stromal and epithelial cells [113,114,115,116].

**Figure 4:**
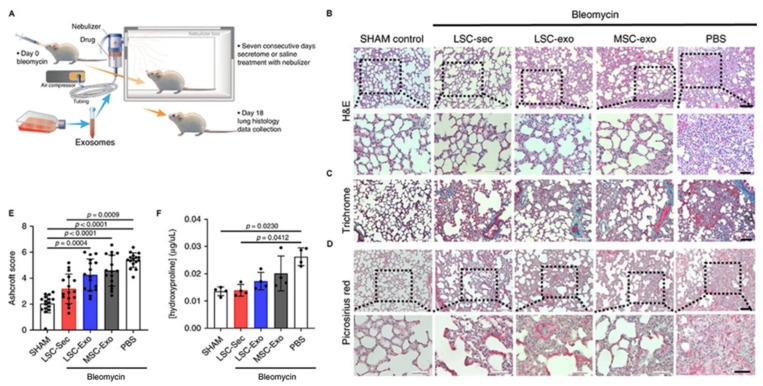
Therapeutic effects of EXO administration in an experimental PF model. (**A**) Schematic illustration of the experimental design for evaluating EXO therapeutic efficacy in pulmonary fibrosis. (**B**–**D**) Representative histological analyses of lung tissue following EXO treatment, including hematoxylin and eosin (H&E), Gomori’s trichrome, and picrosirius red staining, demonstrating attenuation of fibrotic remodeling and collagen deposition. (**E**) Quantitative assessment of pulmonary fibrosis using the Ashcroft scoring system. (**F**) Lung hydroxyproline quantification as a biochemical indicator of collagen accumulation. Reproduced with permission from Dinh et al. [50].

In contrast, EXOs secreted by therapeutic cell populations—such as MSCs, ADSCs, and immunomodulatory M2-like macrophages—exert pronounced anti-fibrotic effects [117,118]. These effects are consistently supported by histological evidence, including reduced collagen deposition and restoration of alveolar architecture observed by H&E, Gomori’s trichrome, and picrosirius red staining, as well as quantitative reductions in Ashcroft scores and lung hydroxyproline content (Figure 4) [50]. Mechanistically, therapeutic EXOs are enriched in miRNAs such as *miR-29*, *miR-let-7d*, *miR-30*, and *miR-186*, which suppress genes involved in ECM synthesis, fibroblast proliferation, and myofibroblast differentiation [119,120]. They also deliver proteins including hepatocyte growth factor, keratinocyte growth factor, and IL-10, thereby promoting epithelial repair, reducing oxidative stress, and fostering anti-inflammatory immune polarization [121,122].

Beyond direct effects on fibroblasts and epithelial cells, EXOs modulate immune responses by regulating alveolar macrophage polarization and regulatory T-cell activity [123,124]. For instance, MSC-derived EXOs induce M2 macrophage polarization via *let-7* family miRNAs, resulting in increased IL-10 production and the suppression of pro-inflammatory cytokines [125,126,127]. These immunomodulatory effects contribute to reduced neutrophil infiltration, improved vascular integrity, and enhanced resolution of lung injury [95]. Collectively, the ability of EXOs to concurrently regulate inflammation, epithelial dysfunction, and matrix remodeling positions them as uniquely suited mediators of fibrotic progression and resolution [1,59].

Importantly, the biological outcome of EXO-mediated signaling is highly context-dependent. Microenvironmental cues, disease stage, cellular origin, and recipient cell type collectively determine whether EXOs promote fibrogenesis or facilitate repair [128,129]. While this heterogeneity complicates therapeutic translation, it also presents an opportunity: rational control of EXO composition through selective isolation or bioengineering may enable the development of more effective and disease-adapted anti-fibrotic therapies. In summary, EXOs orchestrate complex intercellular signaling networks that shape the trajectory of PF, functioning both as drivers of disease pathogenesis and as promising agents for therapeutic intervention [88].

## 3. EXOs as Diagnostic and Prognostic Biomarkers

### 3.1. Biofluid-Derived EXOs in PF

The identification of non-invasive, sensitive, and disease-specific biomarkers remains a major unmet need in the early diagnosis and longitudinal monitoring of PF. In this context, EXOs isolated from accessible biological fluids—including bronchoalveolar lavage fluid (BALF), serum, plasma, sputum, and saliva—have emerged as a promising liquid biopsy platform, as they stably encapsulate molecular information that reflects the physiological and pathological state of their cells of origin [130]. Unlike freely circulating biomarkers, exosomal cargos are selectively sorted and protected by a lipid bilayer, rendering them resistant to enzymatic degradation and particularly suitable for clinical analysis [131,132,133].

Among the exosomal cargos, microRNAs (miRNAs) have attracted the greatest attention as PF-related biomarkers. Although miRNAs are detectable in multiple biofluids, they predominantly circulate in an exosome-associated form, which markedly enhances their stability and reproducibility in clinical samples [131,132,133]. As a result, disease-associated changes in exosomal miRNA profiles provide insights not only into fibrotic mechanisms, but also into diagnostic stratification and therapeutic prediction. In BALF-derived EXOs from patients with IPF, anti-epithelial miRNAs such as *miR-200a-3p*, *miR-200b-3p*, and *miR-141-3p* are consistently downregulated, whereas *miR-320a-3p*, *miR-320b*, and *miR-22-3p* are upregulated, reflecting epithelial injury and fibroblast activation [134].

In parallel, circulating EXOs from IPF patients exhibit elevated levels of profibrotic miRNAs including *miR-21*, *miR-155*, and *miR-199a-5p*, which correlate with disease severity and poor prognosis (Table 2) [135,136,137]. Conversely, reduced levels of anti-fibrotic miRNAs such as the *miR-29* family, *miR-200*, and *let-7d* are associated with increased fibrotic burden and progressive lung function decline [138,139,140].

Beyond miRNAs, exosomal protein cargos have also been explored as complementary biomarkers. Increased expression of EXO-associated proteins such as CD63, TGF-β1, and fibronectin in serum or BALF has been proposed to reflect fibrotic activity and treatment responsiveness, highlighting the potential of multimodal exosomal signatures that integrate both nucleic acid and protein readouts [141,142,143].

BALF-derived EXOs are particularly informative in respiratory diseases because they directly reflect the local pulmonary microenvironment [144]. Recent studies demonstrate that BALF exosomal *miR-22* and *miR-142-3p* can distinguish PF patients from healthy controls and may serve as indicators of alveolar epithelial damage and fibroblast activation [145,146]. Although BALF collection is semi-invasive and less suitable for routine screening, it remains a valuable resource for biomarker discovery and mechanistic validation [147,148,149]. In contrast, serum- and saliva-derived EXOs offer more practical options for repeated sampling and longitudinal monitoring, supporting their potential use in real-world clinical settings [150].

Collectively, these findings highlight the potential of biofluid-derived EXOs as diagnostic and prognostic biomarker candidates in PF. However, most currently reported EXO-based biomarker studies remain limited by preclinical evidence, small patient cohorts, methodological heterogeneity, and the lack of large-scale multicenter validation, which currently restrict their clinical applicability [151,152]. Standardized protocols for vesicle isolation, normalization, and cargo analysis will be essential to improve reproducibility across studies and platforms. Although the integration of exosomal profiling with multi-omics approaches may further improve biomarker discovery and disease monitoring, substantial clinical validation will be required before routine implementation in personalized PF management can be considered [153,154].

### 3.2. Exosomal microRNAs and Their Predictive Value

MicroRNAs (miRNAs) are small non-coding RNAs that regulate gene expression at the post-transcriptional level and play essential roles in cellular differentiation, apoptosis, immune regulation, and fibrotic remodeling [58,155]. When selectively packaged into EXOs, miRNAs are shielded from extracellular degradation, conferring high stability and reproducibility in biological fluids. In PF, accumulating evidence indicates that disease-associated exosomal miRNA signatures are closely linked to disease onset, progression, and treatment responsiveness, positioning them as promising predictive biomarkers rather than passive diagnostic readouts [156,157,158,159,160,161].

**Figure 5:**
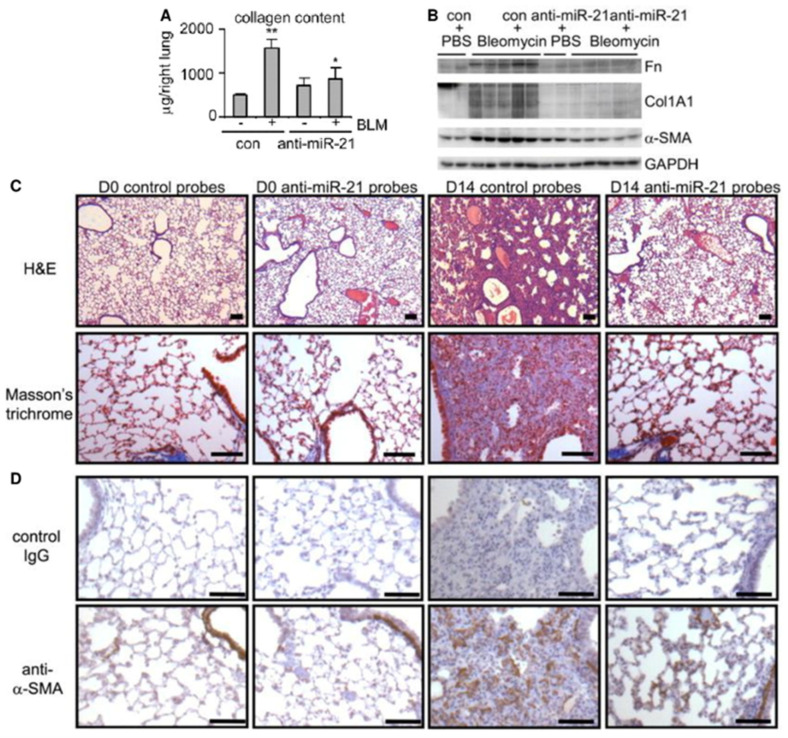
Therapeutic inhibition of exosomal *miR-21* attenuates PF in a bleomycin-induced mouse model. (**A**) Quantification of lung collagen content following treatment with PBS, bleomycin plus control probe, or bleomycin plus *miR-21* antisense probe. (**B**) Representative Western blot analysis of fibronectin, collagen type I alpha 1 (Col1A1), and α-smooth muscle actin (α-SMA) expression in lung tissue. (**C**) Histological evaluation of pulmonary fibrosis by hematoxylin and eosin (H&E) and Masson’s trichrome staining, demonstrating reduced collagen deposition and improved tissue architecture following *miR-21* inhibition. (**D**) Immunohistochemical analysis of α-SMA expression in lung sections, indicating the suppression of myofibroblast activation after *miR-21* antisense treatment. * *p* < 0.05 and ** *p* < 0.01 were considered statistically significant. Reproduced with permission from Liu et al. [164].

Among these candidates, exosomal *miR-21* is the most extensively studied and is consistently associated with fibrotic severity and poor clinical outcomes. Elevated levels of *miR-21*-containing EXOs have been detected in serum and BALF from patients with IPF and correlate with disease progression. Mechanistically, *miR-21* promotes fibrogenesis by suppressing SMAD7, thereby enhancing TGF-β/Smad2/3 signaling, driving myofibroblast differentiation and ECM accumulation [162,163]. Importantly, experimental inhibition of *miR-21* not only reduces collagen deposition but also attenuates fibrotic remodeling in bleomycin-induced PF models, underscoring its functional relevance beyond biomarker association (Figure 5) [164].

In a similar profibrotic context, *miR-199a-5p* is enriched in EXOs derived from fibrotic lungs and circulating biofluids. This miRNA exacerbates fibrotic remodeling by targeting caveolin-1 (CAV1), a negative regulator of TGF-β signaling, thereby amplifying profibrotic signal transduction [137,165,166]. In contrast, several exosomal miRNAs exert protective, anti-fibrotic effects and are consistently downregulated during PF progression. Members of the *miR-29* family, particularly *miR-29a* and *miR-29b*, suppress the expression of collagen synthesis genes such as *COL1A1* and *COL3A1*, and reduced levels of *miR-29*-containing EXOs are associated with increased fibrotic burden and accelerated lung function decline [88]. Additional anti-fibrotic candidates, including *let-7d* and *miR-186*, are similarly diminished in PF-derived EXOs and may reflect a loss of epithelial integrity and unresolved inflammatory signaling [147].

Beyond their mechanistic relevance, exosomal miRNAs exhibit substantial predictive value for clinical decision-making. Baseline serum levels of profibrotic miRNAs such as *miR-21* and *miR-155* have been proposed as predictors of responsiveness to anti-fibrotic therapies, including pirfenidone and nintedanib [167,168]. Moreover, longitudinal monitoring of exosomal miRNA dynamics during treatment has been suggested to provide early indicators of therapeutic efficacy, disease stabilization, or impending relapse, preceding conventional clinical or radiographic changes [169,170].

Despite these promising findings, several challenges must be addressed before exosomal miRNAs can be fully integrated into clinical practice. Technical variability in EXO isolation and miRNA quantification, lack of standardized normalization strategies, and limited validation in large, multicenter cohorts currently constrain their translational applicability [171,172]. Nevertheless, rapid advances in next-generation sequencing, digital PCR, and multi-omics integration are expected to accelerate the clinical implementation of exosomal miRNA-based predictive biomarkers in PF [173,174].

### 3.3. Challenges in Clinical Translation of EXO Biomarkers

Despite the growing interest in exosome (EXO)-based biomarkers for PF, their translation into routine clinical practice remains constrained by a range of technical, biological, and regulatory challenges [1]. Although EXOs offer distinct advantages—including molecular stability in biofluids, cell-type specificity, and the ability to reflect dynamic pathological processes—robust and standardized methodologies are still lacking for their reliable isolation, characterization, and quantitative analysis [1,4].

A major technical limitation lies in the absence of standardized EXO isolation protocols [175,176]. Ultracentrifugation, widely regarded as the gold standard, is labor-intensive, time-consuming, and poorly suited for high-throughput clinical workflows. Alternative approaches, such as polymer-based precipitation kits or size-exclusion chromatography, offer improved scalability but frequently suffer from low purity, co-isolation of non-exosomal EVs, or contamination with protein aggregates [175,177,178]. This platform-dependent variability leads to substantial inconsistencies in exosomal yield, composition, and downstream molecular analyses, thereby compromising reproducibility and cross-study comparability—both of which are essential for biomarker validation.

Combining orthogonal isolation methods—such as size-exclusion chromatography with downstream immunoaffinity capture—substantially improves EXO purity over single-step approaches [179,180]. At the field level, systematic adoption of MISEV guidelines and EV-TRACK-compliant reporting provides a practical and immediately implementable framework for harmonizing isolation practices across research centers [181,182].

Beyond technical considerations, the biological heterogeneity of EXOs presents an additional challenge [183]. In the fibrotic lung microenvironment, EXOs are released from multiple cell types, including alveolar epithelial cells, fibroblasts, endothelial cells, and immune cells, each contributing overlapping yet distinct cargo profiles [1,184]. Without reliable cell-of-origin markers or enrichment strategies, interpreting exosomal signals from complex biofluids such as plasma or BALF remains inherently ambiguous [4]. Furthermore, EXO cargo composition is dynamic and may shift in response to disease stage, therapeutic intervention, or comorbid conditions such as infection or malignancy, potentially confounding biomarker specificity and longitudinal interpretation [162,185].

Cell-type-specific immunoaffinity enrichment—using surface markers such as EpCAM for epithelial-derived or FAP for fibroblast-derived EXOs—enables the targeted isolation of diagnostically relevant subpopulations from complex biofluids [186,187]. Integration of single-vesicle analysis platforms (e.g., nano-flow cytometry) with machine learning-assisted cargo classification further holds promise for resolving EXO heterogeneity and improving biomarker specificity in PF [188,189].

Quantification and normalization of exosomal contents—particularly RNA species—represent another unresolved bottleneck. Currently, there is no consensus on endogenous reference controls for normalizing exosomal miRNA expression, and pre-analytical variables such as sample collection, processing, storage conditions, and freeze–thaw cycles can markedly influence the analytical outcomes [152,190,191]. In addition, the low abundance of certain diagnostically relevant exosomal miRNAs or proteins necessitates ultrasensitive detection platforms, which are not yet widely implemented in standard clinical laboratories [192].

Synthetic spike-in RNA controls and droplet digital PCR (ddPCR)—which enables absolute miRNA quantification independent of endogenous reference genes—offer robust and clinically transferable solutions to current normalization limitations [189,193,194]. For protein-level biomarkers, single-molecule array (SiMoa) technology extends detection sensitivity to the femtomolar range, addressing the analytical gap posed by low-abundance exosomal targets [195].

From a regulatory and translational perspective, EXO-based diagnostics must satisfy stringent criteria for analytical validity, clinical utility, and cost-effectiveness before clinical adoption [196]. Existing biomarker qualification frameworks do not yet fully accommodate the complexity of EVs, underscoring the need for dedicated regulatory guidance tailored to EV-based diagnostics [197]. Addressing these challenges will require coordinated efforts among academia, industry, and regulatory agencies to establish standardized reference materials, proficiency testing schemes, and harmonized clinical trial designs for EXO biomarker validation [197,198,199].

Alignment with established regulatory frameworks—including the FDA BEST resource and EMA qualification opinion process—alongside engagement with international consortia such as the ISEV Biomarker Task Force, provides a structured pathway for advancing EXO diagnostics toward clinical qualification [58,181,200]. Incorporating EXO biomarkers as exploratory co-endpoints within ongoing anti-fibrotic drug trials represents a pragmatic near-term strategy for generating the clinical evidence required for formal regulatory submission [201,202].

Taken together, while exosomal biomarkers hold substantial promise for transforming the diagnosis and monitoring of PF, clinical translation will require a staged approach: beginning with cross-laboratory standardization of isolation and quantification methods, followed by large-scale multicenter validation, and culminating in regulatory-grade biomarker qualification. Sustained interdisciplinary collaboration among researchers, clinicians, and regulatory agencies will be essential to advance EXO-based diagnostics from exploratory tools to actionable clinical assays in fibrotic lung disease.

## 4. EXO-Derived Therapeutics in PF

### 4.1. Natural Sources of Therapeutic EXOs

EXOs derived from naturally occurring cell sources have emerged as promising cell-free therapeutics for PF as they can recapitulate many of the paracrine effects of donor cells while avoiding the safety concerns associated with direct cell transplantation [203,204]. These vesicles inherently carry bioactive cargos—including microRNAs, proteins, lipids, and metabolites—that reflect the regulatory phenotype of their parent cells and enable multifaceted modulation of the fibrotic lung microenvironment [61,205,206,207]. Upon systemic or local administration, natural EXOs exert anti-fibrotic, immunomodulatory, and tissue-reparative effects in multiple preclinical PF models [204,208,209,210,211].

Among the natural sources, mesenchymal stem cell-derived EXOs (MSC-Exos) represent the most extensively investigated and validated platform for PF therapy [212,213,214]. MSC-Exos are enriched in anti-fibrotic microRNAs such as *miR-29* family members, *let-7* family members, *miR-186*, and *miR-129*, which collectively suppress collagen biosynthesis, inhibit TGF-β signaling, and attenuate fibroblast activation [215,216,217,218,219]. Consistent with these mechanisms, administration of MSC-Exos in bleomycin-induced PF models reduces lung fibrosis scores, decreases hydroxyproline content, preserves alveolar architecture, and improves survival [215,220,221].

Beyond their direct effects on fibroblasts and ECM remodeling, MSC-Exos exert potent immunomodulatory functions. They suppress pro-inflammatory cytokine production, promote macrophage polarization toward an anti-inflammatory M2 phenotype, and enhance regulatory T cell responses, thereby mitigating chronic inflammation—a central driver of fibrogenesis [95,222,223,224,225,226,227]. Supporting this concept, MSC-Exo-mediated M2 macrophage polarization has consistently been observed across inflammatory disease models, accompanied by reduced levels of pro-inflammatory cytokines and increased expression of IL-10 [225,228,229].

MSC-Exos also convey anti-fibrotic and cytoprotective signals through growth factors and regulatory proteins inherited from parent MSCs, including hepatocyte growth factor and IL-1 receptor antagonist (IL-1Ra), which inhibit immune cell-mediated lung injury, epithelial–mesenchymal transition (EMT), and collagen deposition [230,231,232,233]. In parallel, MSC-Exos display antioxidant properties by scavenging intracellular reactive oxygen species (ROS), preserving mitochondrial integrity, and enhancing intrinsic antioxidant defenses [234,235]. Collectively, these multifaceted mechanisms contribute to the robust anti-fibrotic efficacy of MSC-Exos observed across pulmonary and extrapulmonary fibrosis models [236].

In addition to MSCs, ADSCs and umbilical cord-derived MSCs have emerged as alternative EXO sources. ADSC-Exos offer advantages such as higher yield, easier harvest, and lower donor-site morbidity while exhibiting comparable anti-fibrotic efficacy in animal models [237,238]. Umbilical cord MSC-derived EXOs display low immunogenicity and high proliferative capacity, making them attractive candidates for allogeneic applications [239,240].

Beyond stem cells, immune cell-derived EXOs—particularly those from alternatively activated M2 macrophages—represent another promising therapeutic avenue [241]. M2-derived EXOs are enriched in anti-inflammatory and reparative cargos such as *miR-223*, *miR-146a*, IL-10, and arginase-1, which facilitate macrophage polarization, suppress neutrophil recruitment, and attenuate oxidative stress [242,243,244,245,246]. Notably, macrophage-derived EXOs carrying *miR-142-3p* have been shown to suppress TGF-β receptor signaling in alveolar epithelial cells and fibroblasts, thereby slowing fibrotic progression [247].

Endothelial progenitor cell-derived EXOs (EPC-Exos) have also demonstrated reparative potential in PF models by delivering pro-angiogenic factors and ECM-regulatory microRNAs that support vascular regeneration, stabilize the alveolar–capillary barrier, and indirectly suppress fibrotic remodeling [248,249,250]. Similarly, epithelial cell-derived EXOs—including those from alveolar type II (ATII) cells, human bronchial epithelial cells (HBECs), and human amniotic epithelial cells (hAECs)—exhibit intrinsic anti-fibrotic properties [251,252,253,254,255,256]. These EXOs inhibit TGF-β-induced myofibroblast differentiation, reduce cellular senescence, and suppress collagen production through coordinated protein and microRNA cargoes [251,252,253,254,255,256].

Given the diversity of therapeutic EXO sources, their translational potential varies considerably in terms of biological activity, scalability, and safety considerations. A comparative overview of the major therapeutic EXO sources relevant to PF therapy is summarized in Table 3.

Despite these advantages, several limitations remain in the therapeutic use of naturally derived EXOs including cargo heterogeneity, donor-to-donor variability, limited yield, and challenges in large-scale biomanufacturing [1,257,258,259]. EXO content and potency are further influenced by donor age, health status, and culture conditions [260,261,262]. To address these challenges, strategies such as standardized donor cell banks, optimized culture systems, and GMP-compliant purification protocols are actively being developed [263]. Collectively, the multimodal functionality, biocompatibility, and favorable safety profile of naturally sourced EXOs position them as compelling candidates for PF therapy, warranting continued validation through rigorous preclinical studies and early-phase clinical trials.

### 4.2. Engineered EXOs for Enhanced Anti-Fibrotic Activity

**Figure 6:**
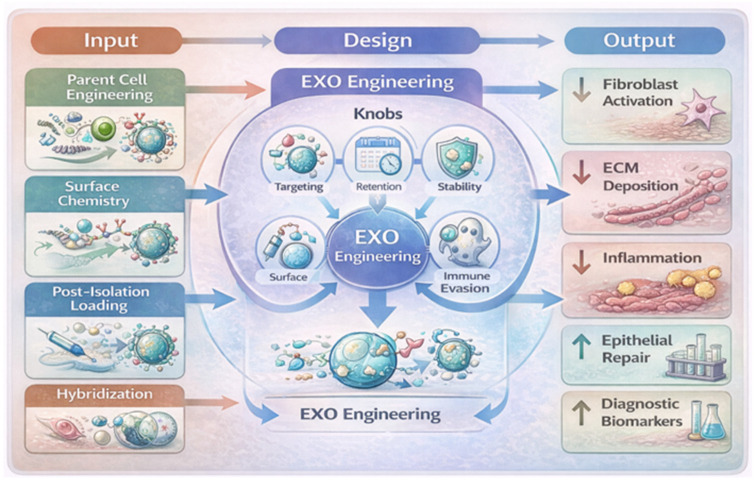
Input–design–output framework for engineering exosomes in pulmonary fibrosis therapy. This schematic illustrates an input–design–output strategy for exosome (EXO) engineering. Input involves loading therapeutic cargos (e.g., miRNAs, proteins, or drugs), design focuses on targeting modification to specific fibrotic lung cells, and output emphasizes formulation and delivery optimization to improve pulmonary retention and therapeutic durability. This framework highlights how engineered EXOs overcome the limitations of native vesicles in targeting and efficacy.

While naturally derived EXOs exhibit intrinsic anti-fibrotic and immunomodulatory properties, their therapeutic performance can be further optimized through bioengineering strategies that precisely control cargo composition, targeting specificity and delivery behavior [264,265]. Engineered EXOs address key limitations of native vesicles—including heterogeneous cargo loading, suboptimal biodistribution, and limited tissue selectivity—by introducing deliberate genetic, molecular, or structural modifications [266]. We therefore discuss engineered EXOs along an “input–design–output” logic: what to load (cargo), where to go (targeting), and how long to stay (formulation) (Figure 6).

#### 4.2.1. Genetic and Cargo Engineering Strategies

One widely explored approach involves the genetic modification of donor cells, such as MSCs, to enrich exosomal cargo with anti-fibrotic molecules [267]. MSCs engineered to overexpress *miR-29b*, *let-7d*, or *miR-181b* produce EXOs with enhanced capacity to suppress collagen synthesis, attenuate TGF-β/Smad signaling, and regulate inflammatory cytokine expression [72,215,268,269]. These engineered EXOs demonstrate superior therapeutic efficacy in preclinical PF models by reducing myofibroblast differentiation and promoting epithelial repair [270].

Complementary to genetic engineering, direct cargo-loading strategies enable the incorporation of exogenous therapeutic agents into pre-isolated EXOs. Techniques such as incubation, electroporation, sonication, extrusion, and freeze–thaw cycling have been employed to load RNAs, peptides, or small-molecule drugs targeting EMT, fibroblast proliferation, and ECM remodeling [266,270]. While passive loading approaches offer simplicity, they suffer from limited encapsulation efficiency, whereas active methods may compromise vesicle integrity or payload stability (Table 4) [271,272].

#### 4.2.2. Surface Engineering and Targeted Delivery

**Figure 7:**
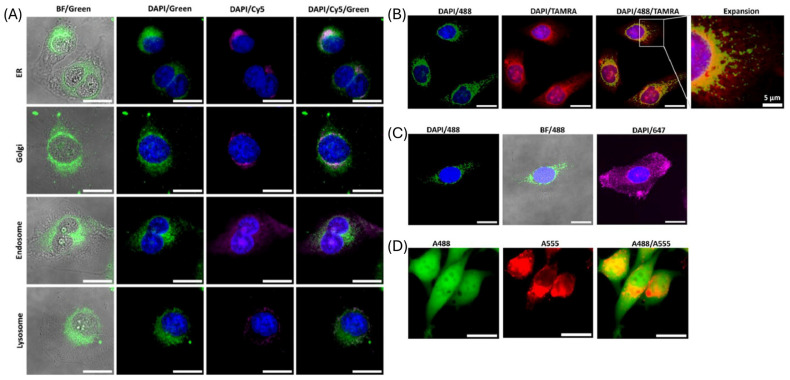
Intracellular trafficking and subcellular localization of enzymatic glycoengineered (EGE) EXOs. (**A**) Confocal fluorescence images showing intracellular trafficking and organelle colocalization of EGE-modified EXOs following cellular uptake. (**B**) Visualization of intracellular delivery of both surface-modified glycans and encapsulated protein cargos. (**C**) Comparative analysis of EXO internalization pathways relative to canonical endocytic uptake. (**D**) Representative fluorescence images confirming efficient cellular internalization and intracellular distribution of glycoengineered EXOs. Reproduced with permission from Kundu et al. [285].

To improve tissue specificity and cellular uptake, surface engineering approaches have been developed, including enzymatic glycoengineering (EGE) and ligand functionalization [284]. Glycoengineering via glycosyltransferases involves attaching an unnatural sugar to the EXO surface using a glycosyltransferase and a modified glycosyl donor [285]. Subsequently, the EXO can be functionalized using click chemistry to link the modified sugar residue (e.g., with an azide) to a tagged molecule (e.g., with an alkyne) and to targeting ligands such as antibodies or peptides that recognize fibrotic tissues [286,287]. In the case of these EXO EGEs, research continues to attach modified sialic acid using sialyltransferases (sial-Ts) due to the complexity of the enzyme. For example, Kundu et al. employed sialyltransferase-mediated incorporation of azide-modified sialic acids onto EXOs, enabling subsequent functionalization with therapeutic proteins and imaging probes [285]. These EGE-modified EXOs exhibited efficient intracellular trafficking and perinuclear localization, suggesting enhanced payload delivery to target cells (Figure 7).

Alternative strategies exploit endogenous EV scaffold proteins. Zheng et al. genetically engineered parent cells to express glycosylated CD63 in combination with fucosyltransferases, generating EXOs with selective affinity for activated endothelial or dendritic cells [284]. In their study, parent cells were genetically engineered to co-express a glycosylation domain (inserted into the large extracellular loop of the scaffold protein CD63) and a corresponding fucosyltransferase (FUT7 or FUT9). This strategy enabled the production of functionalized EVs with high specificity for activated endothelial cells and dendritic cells, respectively.

#### 4.2.3. Hybrid EXO Platforms and Combination Delivery

**Figure 8:**
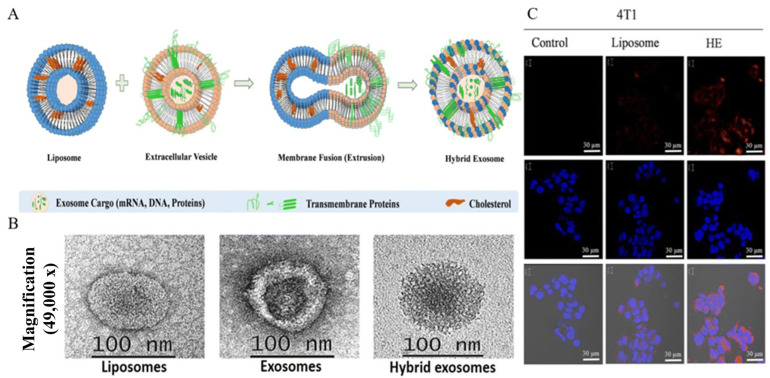
Fabrication and cellular uptake of doxorubicin (DOX)-loaded exosome–liposome hybrid vesicles. (**A**) Schematic illustration of hybrid EXO preparation via membrane fusion between natural EXOs and synthetic liposomes. (**B**) Representative transmission electron microscopy (TEM) images of liposomes, native EXOs, and EXO–liposome hybrid vesicles. (**C**) Confocal fluorescence microscopy images comparing cellular internalization of Rhodamine B-labeled liposomes and hybrid EXOs. Reproduced with permission from Palakurthi et al. [290].

Hybrid EXO–liposome systems represent another powerful engineering strategy, combining the biological advantages of EXOs with the high drug-loading capacity and stability of synthetic liposomes [288,289]. As shown in Figure 8, the fabricated hybrid EXOs not only form slightly larger vesicles compared with liposomes, but also exhibit higher cellular internalization efficiency. These hybrid EXOs maintain a size comparable to that of liposomes while demonstrating enhanced internalization efficiency, thereby improving drug delivery efficiency and ultimately augmenting the therapeutic efficacy of drugs [290].

Hybrid vesicles can be fabricated through incubation, freeze–thaw cycling, or extrusion [291], and have been successfully applied to deliver complex cargos such as CRISPR/Cas9 systems with minimal vesicle damage [292]. Importantly, these systems enable the co-delivery of exosomal cargo and small-molecule anti-fibrotic agents or siRNAs via multiple administration routes, including intravenous, intranasal, or intratracheal delivery [293,294,295].

Beyond hybrid vesicles, engineered EXOs can be integrated into biomaterial-based delivery systems, such as hydrogels or nanoparticle depots, to achieve sustained local release and prolonged retention within fibrotic lung tissue [296,297]. Such composite platforms are particularly attractive for non-invasive administration strategies and for overcoming rapid clearance in the pulmonary microenvironment.

#### 4.2.4. Toward Next-Generation EXO Therapeutics in PF

Looking forward, advances in multi-omics profiling and systems biology are enabling the rational design of personalized EXO therapeutics [281]. By integrating patient-specific transcriptomic and fibrotic signatures, EXOs can be engineered with tailored cargo compositions, targeting ligands, and release kinetics to address inter-individual heterogeneity in PF progression and treatment response [298].

Emerging technologies—including imaging-visible EXOs and stimulus-responsive biomaterials—are further expanding the potential for spatiotemporal control over EXO delivery and activity within fibrotic lungs [299,300]. Collectively, these innovations align with the broader vision of precision nanomedicine for fibrotic diseases.

Despite these advances, significant translational challenges remain, including scalable GMP manufacturing, consistency in engineering protocols, and regulatory classification of hybrid biologics [301,302]. Nevertheless, engineered and hybrid EXOs represent a versatile and modular platform that integrates biological intelligence with nanotechnological control, positioning them at the forefront of next-generation therapeutic strategies for PF.

### 4.3. Delivery Strategies and Routes of Administration

The therapeutic efficacy of EXO-based interventions in PF is governed not only by vesicle bioactivity but also by delivery route and formulation strategy, which critically influence biodistribution, local retention, and pharmacodynamic outcomes [303]. Given the anatomical complexity of the lung and the spatial heterogeneity of fibrotic lesions, rational delivery design is essential to ensure sufficient EXO accumulation at target sites while minimizing off-target exposure [304].

#### 4.3.1. Systemic Versus Local Pulmonary Delivery

Intravenous administration remains the most commonly explored delivery route in preclinical PF studies due to its technical simplicity and systemic accessibility. Owing to the extensive pulmonary capillary network and first-pass filtration effect, IV-injected EXOs preferentially accumulate in the lung [305,306,307]. However, substantial off-target uptake by the liver, spleen, and kidneys often limits lung specificity and necessitates higher dosing [308]. To address this limitation, surface-engineered EXOs bearing targeting ligands for CD44, αvβ3 integrins, or fibrotic ECM components have been developed to enhance selective homing to activated fibroblasts or injured epithelium [304,309,310].

**Figure 9:**
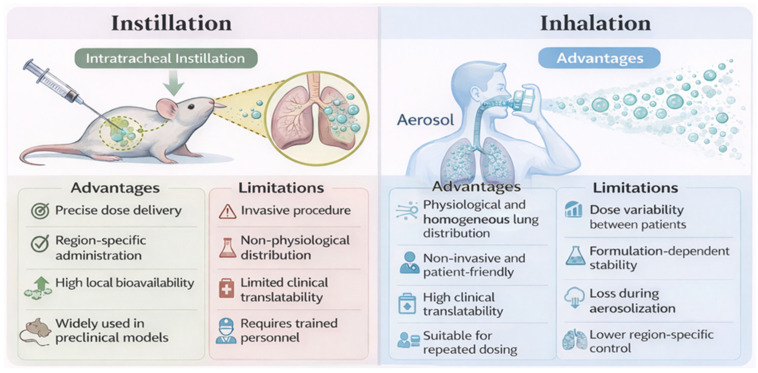
Comparison of intratracheal instillation and inhalation as pulmonary delivery routes for exosome-based therapeutics. Intratracheal instillation allows precise, region-specific dosing and is widely used in preclinical studies, whereas inhalation provides more physiological lung distribution, improved patient compliance, and higher clinical translatability for chronic pulmonary applications.

In contrast, local pulmonary delivery routes—including intratracheal and intranasal administration—enable the direct deposition of EXOs into the alveolar space, bypassing systemic circulation [311]. These approaches significantly improve pulmonary retention and therapeutic efficacy in bleomycin-induced fibrosis models while reducing systemic exposure [50,312]. Importantly, aerosolized or nebulized EXO formulations are under active investigation as non-invasive delivery platforms suitable for repeated or outpatient administration, highlighting their translational potential (Figure 9) [311].

#### 4.3.2. Biomaterial-Assisted and Sustained Delivery Platforms

Beyond direct administration, biomaterial-assisted delivery platforms have been developed to address the rapid clearance and limited retention of EXOs in vivo. Incorporation of EXOs into hydrogels, microparticles, or hybrid matrices enables sustained release, protects vesicles from enzymatic degradation, and prolongs local bioavailability within fibrotic tissues [296,313,314,315,316]. These approaches are particularly relevant in PF, where repeated dosing and long-term modulation of the fibrotic niche are often required.

**Figure 10:**
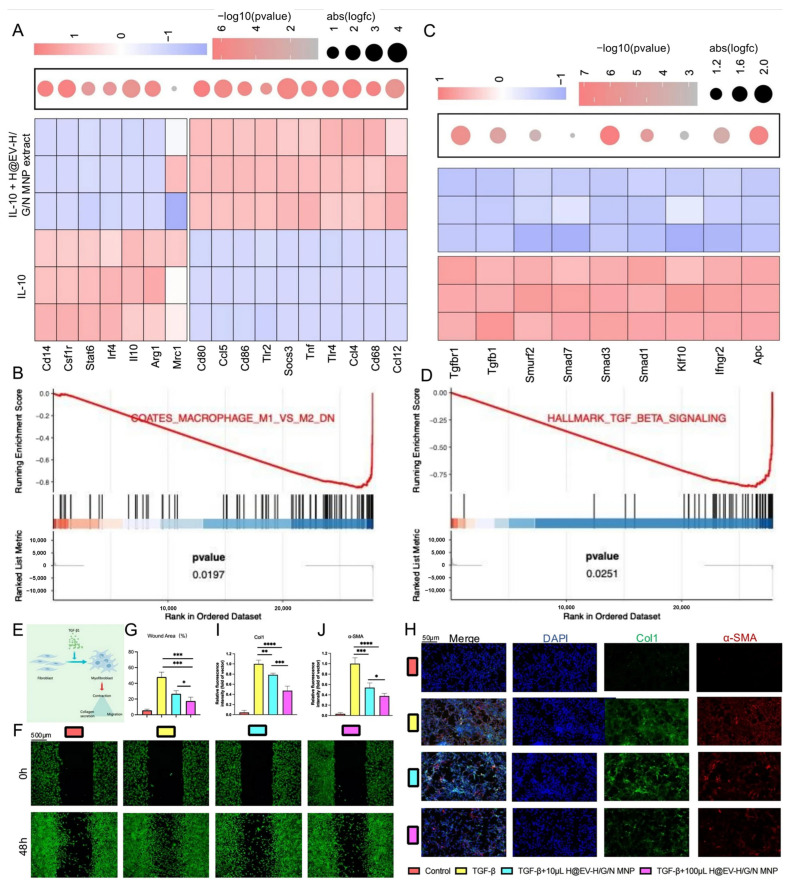
In vitro anti-fibrotic effects of H@EV-H/G/N microneedle patch-derived extracts. (**A**–**D**) Transcriptomic profiling and gene set enrichment analysis demonstrating the modulation of anti-fibrotic and immunoregulatory pathways following treatment. (**E**) Schematic illustration of the proposed anti-fibrotic mechanism of H@EV-H/G/N microneedle patch treatment. (**F**–**H**) Fibroblast migration assays showing reduced cellular motility following treatment. (**I**,**J**) Quantitative analysis of fibrosis-related markers, including collagen type I (Col1) and α-smooth muscle actin (α-SMA), indicating suppression of myofibroblast activation and extracellular matrix production. * *p* < 0.05, ** *p* < 0.01, *** *p* < 0.001, and **** *p* < 0.0001 were considered statistically significant. Reproduced with permission from Song et al. [317].

Although most delivery strategies for PF focus on pulmonary routes, transdermal microneedle-based EXO delivery provides an illustrative example of how sustained, minimally invasive administration can be achieved for chronic fibrotic conditions. In a representative study, Song et al. developed a dissolvable microneedle patch incorporating EXOs, hepatocyte growth factor, and an anti-fibrotic agent, which enabled continuous systemic absorption and prolonged therapeutic exposure [317]. Mechanistically, this platform attenuated fibroblast activation and suppressed TGF-β1/SMAD3 signaling, highlighting how EXO-based combination systems can simultaneously modulate immune responses and fibrotic pathways (Figure 10). While demonstrated in a hepatic fibrosis model, this work underscores generalizable design principles—namely, controlled release, cargo co-delivery, and patient-friendly administration—that are highly relevant to the development of next-generation EXO delivery strategies for PF.

#### 4.3.3. Translational Considerations and Future Directions

Collectively, these delivery strategies illustrate that optimal EXO therapy for PF will likely require a tailored combination of administration route, formulation design, and disease stage. Local pulmonary delivery may be preferable for early or localized disease, whereas systemic or depot-based strategies may better address advanced or diffuse fibrosis. Moreover, integration of EXOs with smart biomaterials—such as stimulus-responsive hydrogels or inhalable dry powders—offers opportunities for the spatiotemporal control of release and improved patient compliance.

As EXO-based therapeutics progress toward clinical translation, systematic evaluation of delivery route-dependent pharmacokinetics, safety, and patient acceptability will be essential. Ultimately, delivery strategy optimization will be as critical as EXO engineering itself in determining the clinical success of EXO-based therapies for PF.

### 4.4. Preclinical and Clinical Evidence Supporting Therapeutic EXOs

Over the past decade, a growing body of preclinical evidence has established the therapeutic potential of exosome (EXO)-based interventions in PF. Studies employing diverse experimental models—including bleomycin-, silica-, and radiation-induced lung injury—have consistently demonstrated that both natural and engineered EXOs attenuate key pathological features of fibrosis, such as excessive ECM deposition, fibroblast activation, and epithelial–mesenchymal transition [1,90].

In bleomycin-induced PF models, systemic or intratracheal administration of MSC-Exos has been shown to reduce fibrotic burden, as evidenced by the decreased expression of profibrotic markers such as α-smooth muscle actin and collagen type I, preservation of alveolar architecture, and improvement in lung function [312,318]. These beneficial effects are mediated in part by exosomal microRNAs, including members of the *miR-29* and *let-7* families, which suppress TGF-β/Smad signaling and collagen biosynthesis, as well as by anti-inflammatory and cytoprotective proteins such as hepatocyte growth factor and TSG-6 [319,320,321]. Importantly, EXOs engineered to enrich these anti-fibrotic cargos have demonstrated superior efficacy in vivo, underscoring the value of rational vesicle modification for therapeutic optimization [319].

Beyond native vesicles, engineered and hybrid EXO platforms have further expanded the therapeutic landscape. Liposome–EXO hybrid systems, for example, have been reported to enhance drug retention, reduce fibrotic remodeling, and improve survival outcomes in experimental PF models [56]. These findings support the concept that multimodal cargo delivery and hybrid formulations can synergistically amplify anti-fibrotic effects beyond those achieved by unmodified EXOs alone.

Across multiple animal studies, EXO-based therapies have repeatedly exhibited favorable safety profiles. They are characterized by low immunogenicity, minimal systemic toxicity, and an absence of off-target organ injury, even following repeated administration [322,323,324]. This safety advantage distinguishes EXOs from cell-based therapies and many synthetic nanocarriers, particularly in the context of chronic lung diseases that may require long-term treatment.

Although clinical trials specifically targeting idiopathic PF remain limited, early human studies provide important proof-of-concept for the feasibility of EXO-based pulmonary therapy. Notably, a Phase I clinical trial (NCT04276987) evaluating aerosolized MSC-derived EXOs in patients with severe COVID-19-associated acute respiratory distress syndrome (ARDS) demonstrated excellent tolerability, absence of dose-limiting toxicity, and improvements in pulmonary inflammation and oxygenation [325,326]. While conducted in an acute lung injury setting, these results establish the safety and practicality of inhaled EXO delivery to the human lung—an essential prerequisite for future clinical translation in fibrotic lung diseases.

Collectively, accumulating preclinical and early clinical evidence supports the therapeutic promise of EXO-based interventions for PF (Table 5). With continued advances in bioengineering, delivery strategies, and regulatory alignment, EXO therapeutics are increasingly positioned to transition from experimental platforms toward clinically viable, cell-free treatments for fibrotic lung disorders.

## 5. Future Perspectives and Translational Challenges

EXO-based therapies have emerged as a promising therapeutic strategy for PF, offering biological specificity, versatile cargo capacity, and unique intercellular communication properties [1]. The convergence of stem cell biology, nanomedicine, and materials science has enabled the development of engineered and hybrid EXOs that address several limitations associated with both conventional anti-fibrotic therapies and native EVs [327]. Despite these advances, the clinical translation of EXO-based therapeutics remains constrained by interconnected scientific, technical, and regulatory challenges. Compared with established nanodelivery platforms such as lipid nanoparticles (LNPs), polymeric nanoparticles, and synthetic extracellular vesicle mimetics, EXOs offer distinct biological advantages, including endogenous biocompatibility and inherent cell-communication capabilities [328,329]. However, these features are counterbalanced by challenges related to biological heterogeneity, limited manufacturing scalability, complex quality control requirements, and regulatory uncertainty. In contrast, synthetic nanoplatforms generally provide superior manufacturing reproducibility, compositional control, and more established translational pathways, although they may face limitations such as immunogenicity, reduced biological targeting fidelity, or lower compatibility with complex biomolecular cargo [330,331]. Accordingly, EXO-based therapeutics should be viewed not as universal replacements, but as complementary nanoplatforms whose clinical utility will depend on specific disease contexts and therapeutic objectives.

One of the most critical barriers is the lack of standardized manufacturing processes for EXO production, encompassing cell source selection, culture conditions, isolation strategies, and quality control parameters [205]. Batch-to-batch variability remains a major concern for clinical-grade EXO preparations, particularly given the sensitivity of EXO cargo to subtle changes in upstream processing [332]. Accordingly, the establishment of Good Manufacturing Practice (GMP)-compliant workflows, together with scalable and reproducible purification technologies such as tangential flow filtration and size-exclusion chromatography, has been identified as a prerequisite for regulatory approval and commercialization (Table 6) [333,334].

In parallel, a comprehensive mechanistic understanding of EXO–target cell interactions within fibrotic lungs is still lacking. Although functional cargos—including miRNAs, long non-coding RNA (lncRNAs), and proteins—have been implicated in anti-fibrotic and immunomodulatory effects, the context-dependent uptake mechanisms, biodistribution kinetics, and downstream signaling pathways of EXOs in PF remain poorly defined [344,345]. Advanced analytical approaches, such as single-cell-resolved profiling, spatial omics, and in vivo EXO tracking, are therefore required to elucidate the spatiotemporal dynamics of EXO activity within the heterogeneous and mechanically altered fibrotic lung microenvironment [335].

Although EXO therapeutics have generally demonstrated favorable safety profiles in preclinical and early clinical studies, potential immunogenicity remains insufficiently explored. While EXOs are often considered less immunogenic than synthetic nanocarriers due to their endogenous origin, immune responses may still arise depending on donor cell source, purification quality, cargo composition, and administration frequency [346,347]. In particular, EXOs derived from allogeneic or xenogeneic sources may carry immunologically active membrane proteins or residual contaminants capable of triggering host immune recognition, especially following repeated administration [348]. Damage to vesicles during isolation or storage—particularly through ultracentrifugation or dead-end filtration—can induce aggregation or membrane disruption, which may trigger unintended immune responses [348,349]. Moreover, the differentiation status of donor cells has been suggested as a determinant of inherent immunogenicity, with less differentiated stem cell sources exhibiting lower immune activation potential [348,349,350]. These findings underscore the importance of both upstream cell selection and downstream processing in minimizing immunogenic risk.

Storage stability represents another practical but critical barrier to EXO translation. Conventional cryopreservation is commonly used for EXO storage, but repeated freeze–thaw cycles may induce vesicle aggregation, membrane disruption, cargo leakage, and progressive loss of biological activity [351,352]. To improve long-term preservation, alternative stabilization strategies such as lyophilization with cryoprotective excipients (e.g., trehalose or sucrose) have been explored to maintain vesicle integrity while facilitating storage, transport, and clinical handling [351,353]. In parallel, encapsulation-based stabilization approaches—including hydrogel immobilization, polymer-assisted formulations, or hybrid carrier integration—may further protect EXOs from structural degradation and enable controlled release under physiologically relevant conditions [346,354]. However, standardized preservation protocols and robust comparability studies remain limited, representing an important translational bottleneck for clinical deployment.

Rapid progress in precision EXO engineering has further expanded the therapeutic opportunities. Surface modification with disease-specific ligands, metabolic glycoengineering, and EXO–liposome hybridization strategies have been developed to enhance targeting specificity, cargo loading efficiency, and delivery control [292,355,356]. Collectively, these advances support the emerging paradigm of potentially personalized anti-fibrotic strategies, in which patient-specific molecular signatures guide EXO design, cargo selection, and administration routes, thereby addressing inter-individual heterogeneity in disease progression and therapeutic response [205,338,339,340].

However, the increasing structural and functional complexity of engineered EXO platforms also introduces substantial translational challenges. Manufacturing scalability remains a major obstacle, as advanced engineering workflows often involve multiple modification and purification steps that may compromise production efficiency and cost-effectiveness. In addition, batch-to-batch reproducibility remains difficult to ensure due to donor cell heterogeneity, cargo variability, and sensitivity to upstream processing conditions. These challenges are further compounded by regulatory uncertainty, as engineered EXOs occupy a complex classification space between biologics, cell-derived therapeutics, and nanomedicine-based delivery systems, necessitating rigorous standardization and quality control frameworks for clinical translation.

Delivery optimization represents another major translational bottleneck. While intravenous and inhalation routes have demonstrated efficacy in preclinical PF models, practical challenges associated with pulmonary administration remain substantial. In particular, aerosolization and nebulization processes may compromise EXO membrane integrity, induce vesicle aggregation, or reduce cargo stability, thereby affecting biological activity and delivery efficiency. Furthermore, the fibrotic lung presents additional barriers, including abnormal mucus accumulation, impaired mucus penetration, mucociliary clearance, enzymatic degradation, and rapid sequestration by alveolar macrophages, all of which may significantly reduce therapeutic retention at target sites [357,358,359,360]. To overcome these limitations, incorporation of EXOs into biomaterial-based carriers—including hydrogels, nanoparticles, and microneedle systems—has been proposed to improve vesicle stability, enhance pulmonary retention, protect cargo from premature degradation, and enable sustained or localized administration strategies [292,336,337].

In addition, technologies for EXO isolation and purification remain insufficiently robust for large-scale production, and these challenges are exacerbated by the inherent heterogeneity of EXO populations [361]. This issue is particularly relevant in PF, where EXOs originate from diverse cell types—including epithelial cells, fibroblasts, endothelial cells, and immune cells—each contributing distinct and sometimes opposing biological signals. Although ultracentrifugation remains widely used due to its simplicity and cost-effectiveness, it is prone to contamination, vesicle aggregation, and sample loss, especially when processing heterogeneous samples [362]. Microfluidic-based isolation platforms enable size- or marker-specific enrichment but are currently limited by device complexity, low throughput, and suboptimal recovery rates [363]. Consequently, no single isolation strategy is sufficient, and combinatorial or application-specific workflows optimized for distinct EXO subpopulations are likely required to achieve clinical-grade consistency [361].

From a regulatory perspective, frameworks governing EXO-based therapeutics are still evolving [339]. EXOs occupy a regulatory gray zone between biologics, cell-derived products, and drug delivery systems, complicating their classification, quality control, and approval pathways [197]. Early and continuous engagement with regulatory authorities to establish harmonized standards for identity, safety, potency, and efficacy—alongside well-designed clinical trials—will therefore be essential to accelerate clinical translation [302,341].

Although direct clinical evidence for EXO-based therapeutics in pulmonary fibrosis remains limited, early clinical studies in related respiratory conditions, including acute respiratory distress syndrome (ARDS), COVID-19-associated lung injury, and inflammatory pulmonary disorders, have provided initial evidence supporting the feasibility and safety of EXO-based interventions [342,343]. These emerging clinical experiences offer important translational insights, but robust disease-specific Phase II/III studies will be required to establish therapeutic efficacy, optimal dosing regimens, and long-term safety in PF patients. Interdisciplinary collaboration across pulmonology, pharmacology, bioengineering, and regulatory science will be critical to translating EXO technology from experimental innovation into approved anti-fibrotic therapy [364].

Looking ahead, accumulating safety data, ongoing clinical trials, and evolving regulatory frameworks suggest that EXO-based therapies are poised to enter the clinical arena. Future success will depend on continued interdisciplinary efforts to standardize manufacturing, deepen mechanistic understanding, and optimize delivery strategies tailored to patient-specific disease profiles. Moreover, convergence with emerging technologies—such as AI-guided EXO design, personalized EXO platforms, and 3D-bioprinted EXO-loaded matrices—has the potential to further expand the therapeutic possibilities. Together, these advances position EXO-based therapeutics as a promising and adaptable platform for precision medicine in PF.

## 6. Conclusions

EXO-based therapeutics have emerged as a transformative cell-free strategy for the treatment of PF. Accumulating preclinical and early clinical evidence demonstrates that both natural and engineered EXOs can effectively suppress fibroblast activation, reduce excessive collagen deposition, modulate aberrant immune responses, and promote the restoration of epithelial homeostasis. These multifaceted therapeutic effects are mediated by diverse bioactive cargos—including miRNAs, lncRNAs, and proteins—that collectively regulate key fibrogenic pathways such as TGF-β/Smad and Wnt/β-catenin while maintaining excellent biocompatibility and low immunogenicity.

Beyond their therapeutic potential, EXOs also represent a promising class of diagnostic and prognostic biomarkers for PF. Their stable, cell-specific molecular cargos—particularly circulating miRNAs such as *miR-21*, *miR-29*, and *let-7d*—closely reflect disease activity, progression, and therapeutic responsiveness. Integration of EXO profiling with multi-omics platforms and liquid biopsy technologies may therefore enable earlier diagnosis, real-time disease monitoring, and more precise patient stratification. From a medicinal chemistry and drug development perspective, EXOs should be viewed as modular and designable nanoplatforms that connect disease biology with controllable delivery, rather than as passive cell-derived vesicles.

Recent advances in EXO engineering, including ligand-based surface functionalization, metabolic glycoengineering, and hybridization with liposomes or biomaterial matrices, have further expanded their therapeutic versatility by improving targeting specificity, cargo stability, and sustained release. In parallel, optimization of delivery routes—most notably intravenous and inhalation-based administration—has enhanced pulmonary accumulation while preserving favorable safety profiles.

Despite these advances, several challenges must be addressed before widespread clinical translation can be achieved. These include the need for scalable GMP-compliant manufacturing, standardized isolation and characterization protocols, and robust potency assays to ensure batch-to-batch consistency. Moreover, a deeper mechanistic understanding of EXO–cell interactions, biodistribution, and fate within the complex fibrotic lung microenvironment remains essential for rational therapeutic design.

Taken together, EXO-based nanotherapeutics represent a promising frontier in PF management, uniquely combining biological intelligence with engineering versatility. Continued progress in bioengineering strategies, delivery technologies, and regulatory standardization is expected to accelerate their transition from experimental platforms to clinically translatable therapeutic strategies for fibrotic lung diseases.

## Figures and Tables

**Table 1:** Representative strategies for EXO engineering and their therapeutic relevance.

Technology	Purpose	Potential Benefits	Reference
Surface modification	Functionalization of exosome membranes with targeting ligands (e.g., peptides, aptamers, antibodies)	Enhanced tissue- and cell-specific targeting, improved cellular uptake, and increased accumulation at diseased sites	[52,57,58]
Cargo loading (RNA or drugs)	Encapsulation or association of therapeutic RNAs and/or small-molecule drugs within EXOs	Protection of labile cargos, improved therapeutic efficacy, reduced systemic toxicity, and feasibility of personalized therapy; enables transport across biological barriers (e.g., BBB)	[58,59]
Fusion with liposomes or nanoparticles	Hybridization of EXOs with synthetic nanocarriers	Combines biological targeting and biocompatibility of EXOs with high loading capacity and physicochemical stability of synthetic systems; allows sustained and controlled release	[55,56,58]
Genetic engineering of parent cells	Genetic modification of donor cells to generate EXOs enriched with predefined therapeutic cargos	Precise and reproducible cargo composition; improved scalability and batch consistency once stable producer cell lines are established	[58]
Click chemistry-based conjugation	Bioorthogonal covalent attachment of functional moieties to exosome surfaces	High specificity and stability of surface functionalization, rapid reaction kinetics under mild conditions, and preservation of exosome bioactivity	[58]

**Table 2:** Representative microRNAs implicated in pulmonary fibrosis pathogenesis, diagnosis, and therapeutic modulation.

microRNA	Source	Study Model/Cohort Size	Fibrotic Role	Diagnostic/Prognostic Significance	Therapeutic Relevance/Target Pathway	Ref.
miR-21	Lung tissue	Radiation-induced mouse PF model (C57BL/6, *n* = 4/group)	Pro-fibrotic	Upregulated in fibrotic lung tissue; potential fibrosis-associated biomarker	Smad7 suppression/TGF-β signaling activation	[135]
miR-155	Macrophage-derived exosomes/Serum	Experimental macrophage–fibroblast fibrosis model; IPF patient serum cohorts	Pro-fibrotic	Elevated serum miR-155 correlates with radiological severity and reduced FVC	Anti-miR-155 therapy attenuates fibrosis; regulates TGF-β/Smad, SOCS1, PI3K/Akt	[136]
miR-199a-5p	Lung tissue/Fibroblast	Bleomycin-induced PF mouse model; IPF patient lungs (94 IPF vs. 83 controls)	Pro-fibrotic	Significantly upregulated in IPF lungs; associated with fibroblastic foci	CAV1 suppression/TGF-β-driven fibroblast activation	[137]
miR-29	Lung tissue/Fibroblast	Bleomycin-induced pulmonary fibrosis mouse model	Anti-fibrotic	Downregulated during fibrosis progression	Restoration suppresses TGF-β/Smad3 signaling, CTGF, collagen deposition	[138]
miR-200	Lung tissue/Epithelium	Human IPF lung tissues (*n* = 34) vs. controls (*n* = 21)	Anti-fibrotic	Downregulation associated with aberrant EMT activation	EMT suppression via ZEB1/ZEB2; modulates TGF-β/Wnt/Notch signaling	[140]

**Table 3:** Comparative translational characteristics of the major therapeutic EXO sources for pulmonary fibrosis.

EXO Source	Key Advantages	Anti-Fibrotic Mechanisms	Relative Yield/Scalability	Safety/Translational Considerations	Limitations
MSC-derived EXOs	Most extensively validated; broad immunomodulatory effects	*miR-29*, *let-7*, *miR-186*, HGF, IL-1Ra, IL-10; suppression of TGF-β signaling	Moderate	Favorable safety profile; strong translational maturity	Donor heterogeneity; batch variability
ADSC-derived EXOs	Easier harvest; higher yield than MSCs; lower donor-site morbidity	MSC-like anti-fibrotic and immunomodulatory activity	High	Good translational feasibility	Donor variability
UC-MSC-derived EXOs	Low immunogenicity; high proliferative capacity; allogeneic potential	Regenerative, anti-inflammatory, and immunomodulatory effects	Moderate–High	Strong clinical attractiveness	Sourcing/standardization
M2 macrophage-derived EXOs	Strong immunomodulation	*miR-223*, *miR-146a*, IL-10, arginase-1; *miR-142-3p*–mediated TGF-β receptor suppression; macrophage polarization	Moderate	Strong biological rationale but manufacturing complexity	M2 phenotype maintenance challenges
EPC-derived EXOs	Supports vascular repair	Pro-angiogenic and ECM modulation	Low–Moderate	Limited evidence	Immature translational stage
Epithelial-derived EXOs	Disease-relevant biology	EMT suppression/epithelial repair	Low	Physiologically relevant but less scalable	Limited therapeutic validation

**Table 4:** Representative strategies for engineering EXOs to enhance therapeutic cargo delivery.

Strategy	Method	Advantages	Disadvantages	Reference
Passive cargo loading	Incubation	Simple procedure; preserves native EXO membrane integrity and surface proteins	Low encapsulation efficiency; limited cargo types	[273,274]
Active cargo loading	Sonication	Higher loading efficiency compared with passive methods	Temporary membrane disruption; potential loss of bioactivity	[275,276]
	Extrusion	Produces vesicles with uniform size distribution; improved cargo incorporation	Alters membrane composition and zeta potential	[277,278]
	Freeze–thaw cycling	Enables formation of EXO–liposome hybrid vesicles; improved loading	Vesicle aggregation and size heterogeneity	[274,279]
	Electroporation	Effective for loading small RNAs (e.g., miRNAs, siRNAs)	Inefficient for large cargos (e.g., mRNA, proteins); optimization required	[277,280]
Surface chemical modification	Click chemistry-based conjugation	High specificity and stability; minimal impact on EXO size and morphology	Requires chemical functionalization steps	[52,281]
Targeting ligand decoration	Antibody conjugation	High cell- or tissue-specific targeting efficiency	Potential immunogenicity; increased manufacturing complexity	[282,283]

**Table 5:** Clinical trials of exosome-based therapies in pulmonary diseases (data retrieved from ClinicalTrials.gov; accessed September 2025.). Clinical trials were identified using the search terms exosome, PF, and exosome/ARDS. This table summarizes key trial characteristics, including disease indication, trial phase, extracellular vesicle (EV) source, route of administration, ClinicalTrials.gov identifier, and study completion date. Although no trials to date have been conducted specifically in IPF, these studies collectively demonstrate the clinical feasibility, safety, and translational momentum of exosome-based therapeutics in lung injury, inflammatory pulmonary disorders, and acute respiratory syndromes.

Indication	Phase	Source of EVs	Injection	ClinicalTrials.gov Identifier	Complete Date
ARDS	II	Bone marrow-derived MSCs	Intravenous (IV)	NCT04493242	22 May 2021
ARDS	III	Bone marrow-derived MSCs	Intravenous (IV)	NCT05354141	31 August 2025
Pulmonary infection (carbapenem-resistant Gram-negative bacteria)	I/II	Human amniotic membrane-derived progenitor cells (haMPCs)	Aerosol inhalation	NCT04544215	1 March 2025
Postinfectious cough	I/II	MSCs	Nebulization (inhalation)	NCT07103980	30 December 2025
SARS-CoV-2-related lung injury	II/III	MSCs	Intravenous (IV)	NCT05216562	30 December 2022
ARDS	I	MSCs	Aerosol inhalation	NCT04276987	31 July 2020

**Table 6:** Key challenges and translational opportunities in exosome-based therapies for PF. This table summarizes the major scientific, technical, and regulatory barriers limiting the clinical translation of EXO-based therapies for PF, together with emerging strategies proposed to address each challenge and enable precision nanomedicine-based interventions.

Challenge/Opportunity	Description	Proposed Solutions	Reference
1. Manufacturing & Standardization	Significant variability in EXO yield, purity, and cargo composition due to differences in cell source, culture conditions, and isolation methods	Development of GMP-compliant manufacturing workflows; adoption of standardized isolation and purification techniques (e.g., tangential flow filtration, size-exclusion chromatography); implementation of validated potency, identity, and release assays	[175,177,178,333,334]
2. Mechanistic Understanding	Incomplete understanding of EXO biodistribution, cellular uptake pathways, and downstream signaling mechanisms within fibrotic lung tissue	Integration of in vivo EXO tracking, single-cell RNA sequencing, spatial transcriptomics, and multimodal imaging to resolve spatiotemporal EXO dynamics	[281,335]
3. Targeting Specificity	Limited and non-specific accumulation of systemically administered EXOs at fibrotic lung lesions	Surface engineering with disease-relevant ligands (e.g., CD38, integrins); metabolic glycoengineering to enhance selective cell and tissue targeting	[52,284]
4. Controlled Delivery	Short circulation half-life and instability of EXOs in the hostile, enzyme-rich fibrotic lung microenvironment	Hybrid vesicle platforms, hydrogel-based depots, and microneedle systems enabling sustained release, local retention, and protection from premature degradation	[292,317,336,337]
5. Personalized Therapeutics	Substantial inter-patient heterogeneity in fibrosis drivers, disease progression, and therapeutic response	Precision EXO design guided by transcriptomic and molecular profiling; modular and programmable cargo-loading platforms	[205,338,339,340]
6. Clinical Translation & Regulation	Absence of well-defined regulatory pathways for EV- and EXO-based therapeutics	Early and continuous engagement with regulatory agencies; establishment of harmonized standards for EXO identity, safety, potency, and efficacy	[302,341]
7. Clinical Evidence Gaps	Limited availability of robust human clinical data demonstrating EXO safety and efficacy in PF	Well-designed Phase II/III clinical trials, particularly employing inhalation-based or localized delivery strategies in IIPF	[342,343]

## Data Availability

No new data were created or analyzed in this study.
